# Providing competency-based family medicine residency training in substance abuse in the new millennium: a model curriculum

**DOI:** 10.1186/1472-6920-10-33

**Published:** 2010-05-11

**Authors:** J Paul Seale, Sylvia Shellenberger, Denice Crowe Clark

**Affiliations:** 1 Department of Family Medicine, Mercer University School of Medicine & Medical Center of Central Georgia, 3780 Eisenhower Parkway, Macon, GA 31206, USA

## Abstract

**Background:**

This article, developed for the Betty Ford Institute Consensus Conference on Graduate Medical Education (December, 2008), presents a model curriculum for Family Medicine residency training in substance abuse.

**Methods:**

The authors reviewed reports of past Family Medicine curriculum development efforts, previously-identified barriers to education in high risk substance use, approaches to overcoming these barriers, and current training guidelines of the Accreditation Council for Graduate Medical Education (ACGME) and their Family Medicine Residency Review Committee. A proposed eight-module curriculum was developed, based on substance abuse competencies defined by Project MAINSTREAM and linked to core competencies defined by the ACGME. The curriculum provides basic training in high risk substance use to all residents, while also addressing current training challenges presented by U.S. work hour regulations, increasing international diversity of Family Medicine resident trainees, and emerging new primary care practice models.

**Results:**

This paper offers a core curriculum, focused on screening, brief intervention and referral to treatment, which can be adapted by residency programs to meet their individual needs. The curriculum encourages direct observation of residents to ensure that core skills are learned and trains residents with several "new skills" that will expand the basket of substance abuse services they will be equipped to provide as they enter practice.

**Conclusions:**

Broad-based implementation of a comprehensive Family Medicine residency curriculum should increase the ability of family physicians to provide basic substance abuse services in a primary care context. Such efforts should be coupled with faculty development initiatives which ensure that sufficient trained faculty are available to teach these concepts and with efforts by major Family Medicine organizations to implement and enforce residency requirements for substance abuse training.

## Background

Despite numerous faculty development initiatives related to primary care substance abuse training over the past three decades [1-10], rates of primary care screening and alcohol counseling remain low [11,12] and many clinicians report a lack of confidence in assessing alcohol use and providing brief advice for alcohol misuse [13]. Among patients referred to substance abuse treatment in the U.S., only 2-10% are referred by physicians [14-16]. Inadequate substance abuse training in post-graduate residency programs is felt to be a major factor contributing to this situation. A 1997 survey regarding Family Medicine substance abuse residency curriculum [17,18] found little change in substance abuse training since 1985 [19]. Of 270 residency directors (58% of a total of 466 residency directors) who completed questionnaires, 203 (75%) reported that their programs offered required substance abuse curriculum, representing an increase of only six programs over the previous 12 years. Median curriculum time among those reporting curriculum was 8 hours [17]. 78% of respondents reported that faculty development on substance abuse was needed at their institutions.

Two recent federally-funded initiatives have created new opportunity for Family Medicine to enhance substance abuse teaching in U.S. residency programs. The first was the Multi-Agency Initiative on Substance Abuse Training and Education for America (Project MAINSTREAM), which created a strategic plan for interdisciplinary faculty development in substance use disorders across 16 different disciplines including medicine, nursing and social work [7-20]. This project defined a set of core clinical competencies for physicians which have now been endorsed by the Office of National Drug Control Policy, the American Medical Association, the American Osteopathic Academy of Addiction Medicine, and the Society of Teachers of Family Medicine (STFM) [21]. The second initiative was the Substance Abuse and Mental Health Services Administration's (SAMHSA) residency training project in Screening, Brief Intervention and Referral to Treatment (SBIRT), which has provided funding to 17 U.S. medical schools to train residents in multiple specialties over a five-year period [22]. Eight of these programs include Family Medicine residency programs. A key to success of such initiatives is the availability of state-of-the-art quality curricula. This article, developed for the December 2008 Betty Ford Institute Consensus Conference on Graduate Medical Education, identifies barriers to effective Family Medicine residency substance abuse education and presents a model curriculum that addresses these barriers.

## Methods

In order to identify barriers to effective substance abuse training and successful approaches to overcoming these barriers, a literature search was conducted in PubMed/MEDLINE for articles discussing substance abuse education in Family Medicine using the MeSH terms 'Family Practice', 'Medical Education', and 'Substance-Related Disorders'. From among the 326 articles identified, authors selected and reviewed all articles related to substance abuse education or training. Criteria for inclusion were articles specifically related to substance abuse education, curriculum or training programs in primary care. Criteria for exclusion were articles from disciplines other than primary care, and education, curriculum or training programs not specific to substance abuse. Additional articles were identified from reference lists of collected articles, resulting in 88 articles relevant to the curriculum. Authors then reviewed current training guidelines of the Accreditation Council for Graduate Medical Education (ACGME) and their Family Medicine Residency Review Committee (RRC), identifying both those general core competencies which Family Medicine residents are expected to attain and existing RRC guidelines specific to substance abuse education. An internet search was conducted to find substance abuse curriculum materials available on the worldwide web. Core competencies for substance abuse training, as defined by Project MAINSTREAM [20], were taken as a starting point for organizing the curriculum. In order to place the proposed curriculum in the context of current challenges in U.S. primary care delivery, publications and educational materials related to the U.S. "Future of Family Medicine" initiative [23] were reviewed, and strategies were included for addressing these challenges. Cost estimates for offering a faculty development fellowship and curriculum dissemination workshops were obtained through discussions with the former Executive Director of STFM, who calculated costs based on two previous faculty development projects sponsored by the STFM in the 1990's, adjusting estimates to 2008 dollars.

Curriculum was developed using traditional design methods, based on existing substance abuse objectives and structured to link with family medicine's core competencies. Steps in the design model included stating the learning needs based on an assessment of gaps in current curricula, determining the objectives, defining the content and organizational plan of the curriculum, describing the learning experiences to take place, and developing an evaluation plan and plans to solicit support for the curriculum [24,25]. The structure resulted in an eight-module curriculum which can be integrated into the educational program of the typical three-year U.S. Family Medicine residency program.

## Results

### Barriers to Effective Substance Abuse Training in Primary Care

The medical literature reveals a long list of clinician barriers to management of substance abusing patients, including stigmatization of substance abusers [26,27], lack of time [28-33], inadequate training [34-37], lack of confidence in their intervention skills [38,39], inadequate office-based assessment and treatment resources [40], lack of referral resources [36,37], confusion as to what constitutes alcohol misuse [27], skepticism about the effectiveness of alcohol counseling [27,32,41-43], fear that asking about drinking may harm the doctor-patient relationship [44-46], hesitancy to intervene unless physical consequences are present [38], low compensation rates [36], failure to implement systems-wide approaches that encourage adherence to screening guidelines [36,42,47], internal barriers or role conflict related to their own substance use [48,49], and concerns that patients will not honestly disclose their drinking practices [32,33,50]. A recent study of primary care encounters in the Veterans' Administration system noted that physicians seemed to have developed comfortable "scripts" for discussing smoking cessation with patients but lacked such scripts for alcohol discussions [51]. Research also indicates that even when substance abuse screening and intervention are done, medical record documentation is poor [39,52,53].

Systems issues within training institutions also generate additional barriers to substance abuse training, including a lack of curriculum time [1,19], inadequate numbers of trained faculty physician teachers and role models [3,48,54,55], failure to provide clinical experience with substance abuse treatment and with recovering patients [48,56], negative impact of clinical training due to exposure to severe chronic relapsing patients and negative attitudes of other physicians and staff [57-60], lack of acceptance of a medical model for addictive disorders [48], limited use of standardized diagnostic instruments [61], lack of uniform standards for evaluating learners' abilities to deal with alcohol-related problems [48], institutional apathy or hostility [19], and differences in learning needs among different medical specialties [62].

In the U.S., three new realities of the twenty-first century also present new challenges and opportunities. First, resident work hour rules have decreased attendance at traditional afternoon conferences at many residency programs by as much as one-third [63]. In addition, increasing numbers of family medicine residents are from other countries [64] where teaching and clinical contact related to substance abuse may be limited or absent. Finally, the changing U.S. primary care practice model, as described in the "Future of Family Medicine" Project of the American Academy of Family Physicians (AAFP), the Association of Family Medicine Residency Directors (AFMRD), and STFM [65], presents numerous elements that new curricula must address including an expanded basket of services, electronic health records, open access visits, group visits, and quality improvement/patient safety.

### Lessons from Past Substance Abuse Training Efforts: What works?

A recent international review of twenty years of substance abuse training efforts by el-Guebaly et al [54] defined several characteristics of effective substance abuse education programs. First, there is growing consensus regarding the effectiveness of combining didactic instruction and interactive educational strategies to teach substance abuse skills [54,66]. Reviews of continuing medical education efforts by Davis, Thomson et al [67,68] found that training interventions that were interactive and encompassed enabling or reinforcement such as role-playing exercises, focus groups, simulated patients, or practice audits with individualized feedback showed increases of 20-40% in positive outcomes. Recent training curricula in two alcohol screening and brief intervention (SBI) projects, the Cutting Back and Healthy Habits projects, demonstrated the ability of combined didactic and interactive skills training to teach alcohol SBI skills effectively [69,70]. The use of training projects with multiple interventions also increases positive outcomes [54]. Davis, Thomson, et al [67,68] found that training efforts using three or more interventions increased positive outcomes by an additional 19% over those using a single intervention. A recent study using virtual reality skills training showed increases in trainees' alcohol screening, intervention and referral skills, indicating that this may be a promising technique for the future [71].

One of the greatest challenges in substance abuse training is that of changing negative attitudes. While evidence-based conclusions in this area are limited, contact with recovering individuals appears to achieve positive results. A Project SAEFP (Substance Abuse Education for Family Physicians) module that included an Alcoholics Anonymous (AA) meeting involving recovering professionals was rated as the project's most powerful teaching components, resulting in increased referrals to both AA and formal treatment programs [5]. El-Guebaly and colleagues [54] suggest that the tendency toward regression in beliefs about role responsibility for substance abusing patients during medical training may be countered by creating a network of positive and experienced clinical faculty who teach and model appropriate attitudes and behaviors. Recent SBI projects affirm that systems changes that provide skills-based training, develop screening, brief intervention, and referral to treatment (SBIRT) procedures and involve multiple staff members can significantly increase substance abuse screening and intervention rates [72,73] and generate long-term increases in residents' confidence in their ability to screen and intervene with at-risk alcohol users [74].

The above literature review suggests application of the following principles, which have guided design of this curriculum: combine didactic and interactive learning strategies, offer regular reinforcement of substance abuse concepts, encourage systems changes that facilitate screening and intervention, provide contact with recovering individuals, and create a network of trained faculty who will teach, reinforce, and model SBIRT skills throughout residents' clinical careers.

## Proposed Family Medicine Substance Abuse Curriculum

### Goal and Objectives

The model curriculum's goal is to equip residents to achieve all of the core substance abuse competencies listed in Table 1. Specific objectives of each individual module are detailed in Additional File 1.

**Table 1:** Core Competencies in Substance Abuse for Family Medicine Residents (adapted from Project MAINSTREAM) [74]

Residents will:	
1	Perform age, gender and culturally appropriate unhealthy substance use screening

2	Effectively assess patients with unhealthy substance use

3	Provide brief interventions to patients with unhealthy substance use

4	Demonstrate effective counseling methods to help prevent unhealthy substance use

5	Refer patients with substance use disorders to treatment settings that provide pharmacotherapy and/or psychosocial counseling for relapse prevention

6	Recognize, treat, or refer co-morbid medical and psychiatric conditions in patients with substance use conditions

7	Refer patients with substance use disorders to appropriate treatment and supportive services

8	Be aware of the ethical and legal issues around physician impairment from substance use and of resources for referring potential impaired colleagues, including employee assistance programs, hospital based committees, and state physician health programs and licensure boards

9	Identify the legal and ethical issues involved in the care of patients with unhealthy substance use

10	Provide pharmacologic withdrawal to patients with substance dependence

11	Provide or refer for treatment for relapse prevention in patients with substance use disorders, both pharmacotherapy and psychosocial counseling

### Content

**Figure 1:**
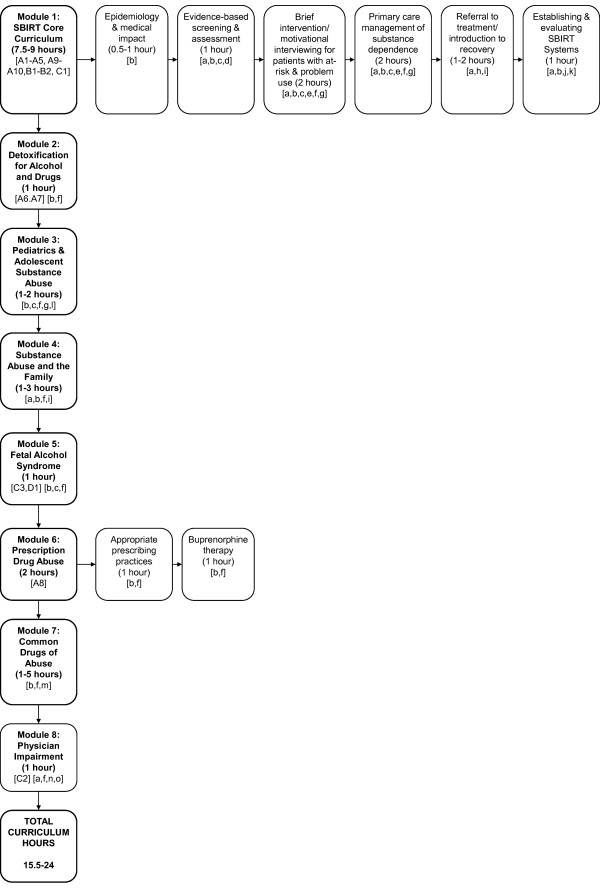
**Flowchart Providing Curriculum Overview, Teaching Strategies, & Online Materials Summary**. **Figure 1 - Teaching Strategies Legend: a **- Training sessions to be conducted live. **b **- Lecture/slide presentation & web module. **c **- Video demonstration. **d **- Role play. **e **- Interview practice with role play & (where feasible) standardized patients &/or recovering patients. **f **- Case-based individual & group discussion. **g **- Feedback. **h **- Panel Discussion. **i **- AA meeting or treatment center visit &/or live family presentation/discussion/interview. **j **- Team-based learning conference with group discussion, team competitions & prizes. **k **- Collaboration with clinic or hospital systems on screening, intervention & documentation strategies. **l **- Optional skills practice. **m **- Case presentation. **n **- Recovering physician presentation with mini-lecture or fact sheet. **o **- Resident orientation lecture. **Figure 1 - Online Material Legend (See also Table 3)**. Mercer University Modules **A1-A10**. Boston University ACT Modules **B1-B2**. Project MAINSTREAM Module **C1-C3**. Project CHOICES Module **D1**

Figure 1 provides an overview of the proposed Family Medicine curriculum, listing all curriculum topics, numbers of curriculum hours, and teaching strategies to be used. Additional File 1 provides detailed information regarding the objectives of the proposed modules and links each objective to specific ACGME competencies. The curriculum consists of eight modules: (1) Screening, Brief Intervention and Referral to Treatment (SBIRT), (2) Detoxification for Alcohol and Drugs, (3) Pediatric and Adolescent Substance Abuse, (4) Substance Abuse and the Family, (5) Fetal Alcohol Syndrome, (6) Prescription Drug Abuse, (7) Common Drugs of Abuse, and (8) Physician Impairment. In the core SBIRT module, composed of six different sections, residents achieve the core competencies of screening, assessment, brief intervention, and referral to treatment. In addition, they receive an introduction to the recovery process and gain an understanding of how to establish and evaluate SBIRT systems in their future practices following graduation. In Module 2, they master the basic skills of detoxification for both alcohol and drugs. Because family physicians work in practices which treat women of childbearing age, children, and adolescents, Modules 3 and 5, Pediatric and Adolescent Substance Abuse and Fetal Alcohol Syndrome, provide skills needed for treating these patient populations. Module 4 addresses Substance Abuse and the Family, recognizing the reality that in primary care, a substance-abusing patient's family member is often the first identified patient. Module 6 addresses the emerging epidemic of prescription drug abuse, teaching guidelines recognition and management of such abuse, appropriate prescribing of mood altering substances, and use of buprenorphine as a primary care tool for managing opioid abuse. Module 7 is a flexible module on drugs of abuse that allows residency programs to focus on common drugs of abuse in their area. Module 8 addresses physician impairment, equipping residents to recognize substance-related impairment and access treatment.

### Educational Methods, Teaching Strategies, and Assessment Components

Figure 1 lists educational and teaching strategies to be employed, including a combination of didactic and experiential learning experiences. All didactic material should be made available in both lecture and web-based format. In an attempt to effect positive attitudinal change, repeated contact with recovering individuals (including recovering physicians), family members and treatment center personnel has been included. Active learning exercises such as role plays, simulated patient interviews, Objective Structured Clinical Exams (OSCE's) [75,76], and virtual patient modules [71] may be utilized to allow residents to practice and develop interview skills and "scripts" to use in patient encounters. Recovering patients may be recruited to serve as simulated patients in sessions where residents practice their resident interview skills [77,78]. Such patients can provide individualized feedback to the residents and share their recovery stories, thus demonstrating that substance use disorders (SUDs) are treatable and creating personal links with 12 step groups or treatment centers. Wherever possible, didactic material should be case-based and focus on substances and scenarios seen in the residency practice. Every attempt should be made to implement an SBIRT system within the residency practice that implements routine alcohol and drug screening, involves other clinic personnel in SBIRT procedures, and supports residents' efforts to make SBIRT a normal part of their day-to-day practice [47]. Novel teaching methods such as Team-Based Learning can be used to teach the residents how to implement SBIRT systems in their future practices [79]. Curricular modules should be reinforced by precepting encounters where multiple faculty communicate the expectation that evidence-based tools be utilized in obtaining substance use histories and interventions and referrals be made for patients with at-risk alcohol use or SUDs.

### Clinical Settings

Substance abuse skills should be modeled and taught across the clinical continuum of care, including the outpatient clinic, emergency department, and inpatient practice. Learning activities at inpatient or residential treatment centers, which have been shown to increase residents' diagnosis and documentation of DSM substance use disorders [80], may be offered as brief or single day activities within rotations such as behavioral science or community medicine, with longer experiences available as electives.

### Resident Assessment Strategies

In order to demonstrate clinical competence in the management of patients with unhealthy substance use, we propose that each resident be required to demonstrate the core SBIRT competencies listed in Table 1 as a graduation requirement. This will require direct observation of resident performance, either through direct or video observation of an inpatient or outpatient encounter, a simulated or standardized patient encounter [81], or screening and intervention exercises included in an OSCE. Unannounced simulated patient visits may also be utilized [42,82]. Ideally, performance should be objectively evaluated using a checklist generated for the OSCE or simulated patient encounter or with a validated assessment instrument such as the Alcohol Interview Performance Evaluation [83]. Where feasible, residents should also participate in at least one family session addressing substance use (SU) issues, which may be included as a component of the residency program's family interview training.

### Faculty

Some previous faculty development efforts [2,10] have employed dissemination models that seek to train individual faculty members to implement curriculum in their residency programs, and STFM's initiative [2] trained only physician faculty, in order to ensure that residents were exposed to physician role models as well as behavioral scientists. Subsequent faculty development programs [7-9,20] have sought to increase impact by training multiple faculty members across departments or disciplines. We have observed that effective substance abuse residency teaching often begins with a faculty "champion" who serves as a leader, coordinator and clinical role model. Faculty champions should seek the residency director's support in communicating the importance of this curricular component. Additionally, substance abuse training is more effective, longer lasting, and more likely to be applied clinically if taught by multiple faculty members; however this goal may be unrealistic for all residency programs. In such cases, collaboration between the faculty physician "champion," the residency's behavioral science faculty, faculty from other area residency programs, or addictionologists from area treatment centers may be used to introduce residents to multiple role models. These efforts should not replace attempts to provide other local residency faculty members with additional substance abuse training. Training opportunities for such faculty include participation in events such as Substance Abuse theme days at national or regional STFM meetings, or participation as faculty mentors at Boston University's Chief Resident Intensive Training (CRIT) program [84]. The STFM Addictions Working Group could assist by organizing Substance Abuse Theme Days and by creating a "resource list" of faculty who would be willing to teach curriculum modules at other programs and/or assist in developing substance abuse curricula.

### Overcoming Training Barriers and Maximizing Opportunities

Additional File 2 demonstrates how the core curriculum, taught using these methods and combined with systems changes advocated above, can address many current training barriers. Lack of time in clinical encounters is addressed by teaching the use of brief validated screening instruments, brief intervention tools and utilizing SBIRT systems which decrease time required by physicians. Stigma regarding substance abuse can be countered by providing repeated contact with recovering individuals and multiple positive faculty role models. Lack of referral resources is addressed by providing contact with recovering individuals who can serve as referral resources, as well contact with treatment center staff. Confusion regarding definitions of alcohol misuse can be clarified by teaching the spectrum of alcohol use disorders. Skepticism regarding treatment effectiveness is countered by evidence-based instruction on the effectiveness of SBIRT techniques and formal substance abuse treatment. Perceived threats to the doctor-patient relationship can be addressed by helping physicians develop "scripts" for addressing substance misuse and allowing them to practice those scripts in repeated clinical simulations. In addition, intervention techniques should be heavily based on principles of motivational interviewing, which teach clinicians to avoid confrontation and "roll with resistance" in the patient encounter [85]. Low compensation rates can be addressed by teaching coding techniques which maximize reimbursement. Strategies for acquiring additional curriculum time include utilizing teaching modules that fit into a variety of teaching contexts (e.g., morning report, noon conference, Grand Rounds, case discussions, etc.). Because total residency curriculum time is limited and faculty often encounter difficulty negotiating hours dedicated specifically to substance abuse, additional substance abuse content can be taught by linking with other curricular areas which teach topics that include substance abuse content (see Table 2).

**Table 2:** Other Residency Topic Areas where Substance Abuse Concepts May Be Taught

Family life cycle stages and challenges	Population medicine
Adolescent medicine	Interpreting the medical literature
Parent education	US Preventive Services Task Force
Family interviewing	guidelines
Motivational interviewing	Health disparities
Patient-centered interviewing	Culturally-sensitive care
Preventive medicine	Management of patients' chronic illnesses
Case-based discussions (Balint-type group discussions)	Mental health assessment
	Practice management
Community medicine	Family violence curriculum
Obstetrics	Geriatrics

This curriculum has been carefully designed to address some of the new challenges presented by current work hour regulations and the emerging model of Family Medicine. In order to address challenges of decreased conference attendance by residents due to work hour regulations, the curriculum's core content should be prepared in web-based modules which can be completed by residents unable to attend conferences, and completion of 80-100% of such modules should be included as a graduation requirement. The new model of Family Medicine encourages each practice to offer a "basket of services." This curriculum offers residents a number of "new skills" that many current primary care practices do not offer which add value and potential practice income for graduating residents who practice in fee-for-service settings. These include screening and brief intervention, which are now reimbursable by Medicare, many private insurers, and Medicaid in some states; office detoxification for alcohol dependence; and office medical management of alcohol dependence and opioid dependence using medications such as naltrexone, acamprosate, and buprenorphine. The electronic health record (EHR) can be used to streamline SBIRT procedures by programming periodic routine alcohol and drug screening for all patients; automatically linking to assessment instruments for patients with positive alcohol and drug screens; providing click-and-print intervention guides, patient education materials, and treatment resource lists; coding for optimized billing for services such as substance abuse screening, brief intervention, and buprenorphine induction; and facilitating improved care of patients with SUDs by appropriately adding SUD diagnoses to problem lists. Group visits can be utilized for patients on medication management for alcohol and opioid dependence, who also benefit from adding counseling interventions to their medical visits, and for patients with stress symptoms or medical problems due to other family members' substance use. Quality improvement initiatives can be developed to document and improve screening and intervention rates. Patient safety features such as drug interaction programs can be used to avoid potentially dangerous interactions such as acetaminophen use with alcohol use disorders and benzodiazepine use with buprenorphine. International medical graduates with limited previous substance abuse experience may require additional training, but may also demonstrate fewer negative attitudes toward patients and readily embrace an active role in their treatment. Faculty may utilize Module 1a to initiate discussions regarding the epidemiology of SUDs in residents' countries of origin, as well as typical attitudes toward patients with SUDs. Likewise Modules 1b and 1c should include training in cultural competency, so that residents understand how screening, assessment and treatment are carried out in a way that is culturally sensitive with all patients.

### Program Evaluation Methods

The ACGME recommends using aggregate data to evaluate the efficacy of residency programs [86]. This same method of using aggregate data can be employed to assess specific parts of the curriculum, such as training in SBIRT. Examples of the types of data to aggregate are resident evaluations of the SBIRT teaching segments, faculty evaluations of the SBIRT teaching segments, and residents' attainment of the specific goals and objectives of the SBIRT educational experience. If one piece of data reveals problems with teaching or performance, for example a group of residents giving poor ratings on evaluations of SBIRT lectures and practice, this information can lead to changes in teaching format or content. As another example, if half of the group of residents performs poorly on OSCE evaluations and the other half performs well, reasons can be explored for the differential performance and strategies employed to improve performance of the poorer performing group. We recommend that aggregate data be reviewed after each new SBIRT teaching segment and yearly with established SBIRT curriculum.

### Implementation and Dissemination Plan

In order to achieve its intended impact in residency programs across the U.S., this curriculum must be combined with other interventions which address important systems barriers to effective substance abuse teaching. A faculty development initiative, modeled after the 1987-1988 STFM/NIAAA (National Institute on Alcohol Abuse and Alcoholism)/NIDA (National Institute on Drug Abuse) program [2], is needed to train a new cadre of faculty as model substance abuse teachers. Currently very few Family Medicine training programs offer substance abuse fellowships, and fellowships which exist in other departments and disciplines are heavily oriented toward research, rather than teaching. Because 85% of Family Medicine residencies are community-based, efforts should be made to recruit and enroll significant numbers of trainees from community-based residency programs. Funding from federal agencies such as NIAAA, NIDA, and HRSA (Health Resources and Services Administration) should be sought for such an effort, perhaps through a contract similar to the previous STFM contract or the National Institutes of Health's R25 education grant cycle. This fellowship initiative should be followed by a series of curriculum dissemination workshops, modeled after Project SAEFP, in order to produce a curriculum training resource and train the faculty champions needed to teach substance abuse at the 458 Family Medicine residency programs. Special efforts should be made to recruit faculty from the 67 residency programs who reported no substance abuse teaching in the 1997 survey and from the roughly 200 residency programs who did not respond at all to the survey [17]. Substance Abuse theme days at national and regional STFM meetings should be organized on an ongoing basis. Finally, regional "Centers of Excellence" in substance abuse teaching should be established to provide long-term continuity in expanding substance abuse training.

Leadership for national efforts to increase substance abuse teaching should include a working group with representatives from the key national Family Medicine organizations such as STFM, AFMRD, AAFP, and the Family Medicine Residency Review Committee (RRC) of the ACGME. In order to increase motivation among programs which currently offer little or no substance abuse teaching, RRC requirements for teaching substance abuse should be strengthened and monitored at the RRC's periodic site visits. The current RRC requirement for substance abuse training is a brief comment tucked in the middle of a paragraph describing various curricular areas in Human Behavior and Mental Health that must be taught [87]. A designation of substance abuse as its own curricular area, separate from Human Behavior and Mental Health, should be sought. In the same way that curricular areas such as maternity care and gynecology are currently required and monitored, the offering of substance abuse curriculum should be required and monitored, and programs should be cited if they are not in compliance [88]. Programs should no longer be allowed to omit the teaching of substance abuse from their residency programs, but rather should have clearly identified faculty who are responsible for teaching this material, under the supervision of the residency director.

### Costs

To develop and implement a model curriculum will incur both national and local costs. The most costly portions of this implementation plan are sponsorship of a national faculty development fellowship initiative to train ten new fellows, which we estimate to cost $500,000; the cost of a curriculum development and dissemination project, which we estimate to cost an additional $2.5 million over five years to train 240 faculty members; and the expense of developing "Centers of Excellence" (no cost estimate available at this time). Support for these programs should be actively sought with federal agencies including NIAAA, NIDA, HRSA, Center for Substance Abuse Prevention (CSAP), and the Center for Substance Abuse Treatment (CSAT).

Local curriculum implementation costs in terms of money and time for a model program are modest, consisting primarily of (1) the faculty time needed to learn and teach the curriculum, and (2) evaluation of learning. National faculty development efforts would offer local faculty the opportunity to learn and teach substance abuse curriculum. Sponsorship may be offered jointly between the government or other organizational sponsors and the local residency program. Most curricular materials are available free of charge via the internet (see Table 3; materials also listed in Figure 1). Once trained, teaching faculty members already employed by the local residency could dedicate 15 1/2-24 curriculum hours of their time to instruction. The most costly portion of the proposed curriculum is the use of standardized patient exercises or OSCE's, which assist learners in acquiring interview skills and in evaluative feedback, but this form of learning and evaluation is not essential to the curriculum. A less costly alternative is use of simulated patient exercises with recovering individuals within local communities, who typically do not charge for participation and welcome the opportunity to spread the message of recovery.

**Table 3:** Curriculum Resources Available on the Internet Specific sections identified from these websites that are particularly relevant are listed below

**A. Mercer University School of Medicine **http://medicine.mercer.edu/sbi	
**A1**	Modules 1, 2, 3, and 4 of Mercer's Initial training http://medicine.mercer.edu/files/sbi_alocholtrainenglish_010407.ppt

**A2**	Mercer's Participant and trainer's guides covering modules 1-4 available at http://medicine.mercer.edu/Introduction/Anesthesiology/Introduction/sbi/sbi_training

**A3**	Mercer's SBI videos for Initial training. Videos 1-5 http://medicine.mercer.edu/Introduction/Anesthesiology/Introduction/sbi/sbi_training

**A4**	Mercer's lecture and cases related to "Evidence base for low-risk drinking guidelines" http://medicine.mercer.edu/sbi_sessionone

**A5**	Mercer's lecture and cases related to "Medication use for treatment of alcohol abuse" http://medicine.mercer.edu/sbi_sessiontwo

**A6**	Mercer's module on "Alcohol withdrawal and delirium tremens". http://medicine.mercer.edu/files/sbi_alcoholwithdrawl_2005.ppt

**A7**	Mercer's module on "Outpatient detoxification." http://medicine.mercer.edu/files/sbi_outpatientalcoholdetox.ppt

**A8**	Mercer's teaching module on "Guidelines for prescribing mood-altering drugs" http://medicine.mercer.edu/sbi_challenges

**A9**	Mercer's "Primary Care Implementation Plan Decision-Making Guide" http://medicine.mercer.edu/The%20Georgia/Texas%20%22Improving%20Brief%20Intervention%22%20Project/sbi_guide

**A10**	Mercer's lecture on "Implementing alcohol SBI in your own practice" http://medicine.mercer.edu/sbi_sessionthree

**B. Boston University's Alcohol Clinical Training Project (ACT) **http://www.bu.edu/act/index.html	

**B1**	Boston University's core curriculum on "Helping patients who drink too much: A web-based curriculum for primary care physicians" http://www.bu.edu/act/mdalcoholtraining/slides/index.html

**B2**	Boston University's related curriculum materials on "Health Disparities and Cultural Competency", and "Pharmacotherapy". http://www.bu.edu/act/mdalcoholtraining/related_curricula.html

**C. Project MAINSTREAM **http://www.projectmainstream.net/projectmainstream.asp?cid = 21	

**C1**	Project MAINSTREAM's lecture on "Alcohol and drug dependence: Comparisons to other chronic medical conditions. http://www.projectmainstream.net/projectmainstream.asp?cid=1298

**C2**	Project MAINSTREAM's lecture "Substance use disorders in physicians" http://www.projectmainstream.net/newsfiles/physician.ppt

**C3**	Project's MAINSTREAM's lectures on pregnancy and SUD http://www.projectmainstream.net/projectmainstream.asp?cid=855

**D. Project CHOICES**	

**D1**	Project CHOICES for women at risk for alcohol-exposed pregnancies. http://www.cdc.gov/ncbddd/fasd/research-preventing.html

## Discussion

Though somewhat limited by the absence of specific evaluation data, this curriculum integrates components of previous effective education programs, while also offering several new and unique elements. It builds on lessons from past educational research and curriculum projects by adopting the substance abuse competencies defined by Project MAINSTREAM [74] and providing a comprehensive review of key topics, similar to Project SAEFP [1]. It integrates effective training approaches identified in previous studies such as combining didactics and interactive activities in skills training [54,69,70]; utilizing active learning strategies such as OSCE's, standardized patient interviews, team learning, and virtual patient exercises [71,75,76,79,81]; using multiple teaching interventions to reinforce concepts [54,79]; and creating a network of clinical mentors and role models [54]. Unique features of this curriculum include the fact that it is mapped to the core ACGME competencies and provides ways to address new challenges presented by U.S. resident work hour rules, racial and ethnic diversity among residents, and an emerging new model of primary care.

## Conclusions

In response to the clinical and training challenges facing U.S. Family Medicine residencies in the 21^st ^century, we propose a curriculum approach that would train residents with a combination of didactic and interactive learning strategies, offer periodic reinforcement of substance abuse concepts, encourage systems changes that facilitate screening and intervention, provide contact with recovering individuals, and document clinical competency by direct observation of resident skills. Implementation of such a curriculum on a broad scale would require a new faculty development initiative to train a new cadre of national substance abuse teaching experts; a series of dissemination workshops to create a network of trained faculty who could teach, reinforce, and model SBIRT skills throughout residents' clinical careers; and development of centers of teaching excellence to provide long-term continuity. Efforts could be greatly enhanced by networking with national Family Medicine organizations to implement and enforce clear RRC requirements for substance abuse curriculum training.

## Abbreviations

Abbreviations found within the text, tables, figures and supporting materials are as follows: AA: Alcoholics Anonymous; AAFP: American Academy of Family Physicians; ACGME: Accreditation Council for Graduate Medical Education; AFMRD: Association of Family Medicine Residency Directors; AMERSA: The Association for Medical Education and Research in Substance Abuse; CRIT: Boston University's Chief Resident Intensive Training; CSAP: Center for Substance Abuse Prevention; CSAT: Center for Substance Abuse Treatment; EHR: Electronic Health Record; HRSA: Health Resources and Services Administration; I&CS: Interpersonal and Communication Skills; MERF: The Medical Education and Research Foundation for the Treatment of Alcoholism and Drug Dependencies; MK: Medical Knowledge; NIAAA: National Institute on Alcohol Abuse and Alcoholism; NIDA: National Institute on Drug Abuse; OSCE: Objective Structured Clinical Exams; PBL&I: Practice-Based Learning and Improvement; PC: Patient Care; PRO: Professionalism; Project MAINSTREAM: Multi-Agency Initiative on Substance Abuse Training and Education for America; R25: National Institutes of Health Research Education Grant Cycle; RRC: Family Medicine Residency Review Committee; SAMSHA: Substance Abuse and Mental Health Services Administration; SAEFP: Substance Abuse Education for Family Physicians; SBIRT: Screening, Brief Intervention and Referral to Treatment; SBI: screening and brief intervention; SBP: Systems-Based Practice; STFM: Society of Teachers of Family Medicine; SU: Substance Use; SUD: Substance Use Disorders; TWEAK: a validated alcohol screening test for pregnant women.

## Competing interests

The authors declare that they have no competing interests.

## Authors' contributions

SS created the curriculum outline and tables, JPS created the introduction and discussion, DCC assisted SS and JPS with the literature search and created Additional File 1, and all three authors together completed and edited the final draft. All authors read and approved the final draft.

## Pre-publication history

The pre-publication history for this paper can be accessed here:

http://www.biomedcentral.com/1472-6920/10/33/prepub

## Supplementary Material

Additional file 1 **Curriculum Objectives and ACGME Competencies**. Legend of ACGME Competencies: Patient Care - PC, Medical Knowledge - MK, Practice-based learning and Improvement - PBL&I, Interpersonal and Communication Skills - I&CS, Professionalism - PRO, and Systems-based Practice - SBP. Click here for file

Additional file 2 Strategies for Overcoming Substance Abuse Training Barriers. Click here for file
