# Supplementary material for: Providing competency-based family medicine residency training in substance abuse in the new millennium: a model curriculum
Source: BMC Med Educ. 2010 May 11;10:33. doi: 10.1186/1472-6920-10-33 (PMC2885404; doi:10.1186/1472-6920-10-33)
Supplement: Additional file 1 — Curriculum Objectives and ACGME Competencies. Legend of ACGME Competencies: Patient Care - PC, Medical Knowledge - MK, Practice-based learning and Improvement - PBL&I, Interpersonal and Communication Skills - I&CS, Professionalism - PRO, and Systems-based Practice - SBP. [file 1472-6920-10-33-S1.DOC]

| **Additional File 1 - Curriculum Objectives and ACGME Competencies**  **Module 1. Core curriculum on Screening, Brief Intervention and Referral for Treatment (8-9 ½ curriculum hours).** | |
| --- | --- |
| **a.** | **Epidemiology and medical impact of SUDs. ½ -1 hour. Lecture/web module.** |
|  | **Learning Objectives:** Upon completion of this module, the learner will be able to: |
|  | Define SUDs as a leading cause of preventable death in the US. (MK) |
|  | Define substance use, substance abuse, and substance dependence and their prevalence in family medicine and the US population. (MK) |
|  | Define low-risk, at-risk and dependent drinking and their prevalence in primary care. (MK) |
|  | Describe health, familial and societal consequences of SUDs such as liver disease, cardiovascular disease, cancer, HIV infection, pancreatic disease, sexual dysfunction, organic brain disease, fetal alcohol syndrome, family violence, MVAs, injuries, depression, suicide, and homicide (MK). |
|  | Identify medical complications of SUDs commonly seen in the local practice setting. (MK) |
| **b.** | **Evidence based screening and assessment, including identification and education of low-risk patients, reinforcement of safe drinking limits. 1 hour live. Lecture with video demonstration, role play.** |
|  | **Learning Objectives:** Upon completion of this module, the learner will be able to: |
|  | Obtain a patient history with attention to current and past use of substances (alcohol, illicit drugs, misuse of prescription medications). (PC) |
|  | Score, interpret, and provide feedback to patients about their substance use using evidence-based pre-screening and screening instruments. (PC) |
|  | Demonstrate ways to respond to patients’ questions about healthy drinking limits. (PC) |
|  | Describe the findings of clinical trials demonstrating the effectiveness of screening and brief interventions by primary care clinicians to reduce SUDs among hazardous and harmful users. (PBL&I)  Based on the literature, describe the cost savings resulting from brief interventions. (PBL&I) |
|  | Describe recommendations of the US Preventive Services Task Force for screening and brief intervention for SUDs. (PBL&I) |
|  | Summarize results of studies indicating that trained clinicians increase their self-confidence and self-efficacy in performing brief interventions and their rates of conducing brief interventions. (PBL&I) |
|  | (In live conference setting) Demonstrate how to perform screening and assessment for substance misuse in a brief role play |
| **c.** | **Brief Intervention using Motivational Interviewing techniques for patients with at-risk and problem substance use (include role play, skills practice). 2 hours live. Lecture, video (or live) demonstration, interview practice, and feedback.** |
|  | **Learning Objectives:** Upon completion of this module, the learner will be able to: |
|  | Use motivational interviewing techniques (asking open-ended questions, developing discrepancy, expressing empathy, avoiding argumentation, rolling with resistance and supporting patient self-efficacy), assist patients in identifying problems their SU can cause and reasons they may want to quit or cut back. (PC) |
|  | Demonstrate an approach to a patient with a SUD that shows respect and compassion for the patient, and sensitivity to the patient’s cultural background, age, gender, and disability. (PRO) |
|  | Provide feedback regarding the patient’s substance use and consequences or potential consequences. (PC) |
|  | Provide clear advice to reduce or stop substance use. (PC) |
|  | Identify patients’ stages of readiness to change. (I&CS) |
|  | Demonstrate the use of open-ended questions and active / reflective listening to encourage patients to discuss the use of and consequences of SUDs in themselves and their families. (I&CS) |
|  | Demonstrate the occasional use of closed-focused questions to determine specific consequences of SUDs such as drinking or drug use while driving, occurrence of violence, or health consequences. (I&CS) |
|  | Demonstrate ways of affirming and supporting the SUDs patients’ thoughts about or efforts to change their use of substances. (I&CS) |
|  | From time to time during the conversation and at the conclusion, summarize the patients’ statements including ambivalence they have expressed about changing. Highlight their thoughts about why they may want to make changes. (I&CS) |
|  | For patients who are ready to reduce substance use, contract with the patient to reduce substance use to a specified amount. (PC) |
| **d.** | **Management of substance dependence in the primary care setting using brief intervention, patient education, and a menu of options including pharmacotherapy, self-help groups and referral to treatment. 2 hours live. Lecture, video (or live) demonstration, interview practice, feedback.** |
|  | **Learning Objectives:** Upon completion of this module, the learner will be able to: |
|  | Describe the importance of genetic predisposition of SUDs and practice sharing this information with patients. (MK, PC) |
|  | Identify medications currently used in the care of patients with substance dependence, their indications and limitations for use, their major side effects and their drug interactions. (MK) |
|  | Demonstrate the use of contracts with patients for cutting back their use of substances with attention to aspects of both patient and physician responsibility. (PC) |
|  | Offer patients with alcohol or drug dependence or in need of relapse prevention a menu of therapeutic options such as pharmacotherapy, self-help groups, and/or referral to treatment. (PC) |
|  | Negotiate a plan for patient referral and/or outpatient medical management using pharmacotherapy and ongoing counseling for a patient with alcohol or drug dependence. (PC) |
|  | Recognize, treat and/or refer co-morbid medical and psychiatric conditions in patients with SUDs. (PC) |
|  | Describe how patient confidentiality of medical records will be protected in accordance with guidelines of both HIPAA and 42CFR. (PRO) |
| **e.** | **Linkages to treatment and the recovery community, including contacts with recovering patients and treatment center personnel. 1-2 hours live.** **Panel discussion, presentation by recovering individuals and/or visit to AA meeting or treatment center.** |
|  | **Learning Objectives:** Upon completion of this module, the learner will be able to: |
|  | Explain the role of substance abuse treatment centers, AA, NA, and other community-based self-help groups in the treatment of SUDs and in relapse prevention. (PC) |
|  | Engage in conversations with recovering individuals to hear their advice on how to approach patients with substance dependence and how to encourage patient participation in self-help groups. (PC) |
|  | Engage in conversations with treatment specialists to discover their methods for evaluation, models of treatment, and preferred modes of collaboration. (PC) |
|  | Describe the specialized SUDs treatment programs available in the community and specialized services needed but not available. (SBP). |
| **f.** | **Systems of care: how to establish and evaluate an SBIRT system in the resident’s clinic or hospital and get reimbursed. 1 hour, preferably live with graduating residents. Team-based learning conference or lecture/web module.** |
|  | **Learning Objectives:** Upon completion of this module, the learner will be able to: |
|  | Critique the residency program’s and/or hospital’s system for identifying substance use level of patients and the mechanisms for intervention and referral at the site. (SBP) |
|  | Develop a strategy for SBIRT implementation by a resident graduate joining a new practice. (SBP) |
|  | Demonstrate familiarity with an implementation guide which can be used to design a system for the resident’s future practice with attention to the following: identification of substance use of patients, identification of family consequences of SUDs, roles for nurses or triage personnel and clinicians in conducting screening and/or brief interventions, methods for coding and documentation of intervention and plan, resources for patients or family members with SUDs. (SBP) |
|  | Outline methods for maximizing reimbursement for provision of SBIRT services. (SBP) |
|  | Describe how a Quality Improvement approach could be used to increase rates of screening, intervention, and reimbursement. (SBP) |
| **Module 2. Detoxification for alcohol and drugs. 1 hour. Lecture or web module.** | |
|  | **Learning Objectives:** Upon completion of this module, the learner will be able to: |
|  | Identify signs and symptoms of alcohol or drug withdrawal. (PC) |
|  | Identify methods of treating alcohol or drug withdrawal in the inpatient setting. (PC) |
|  | Identify methods for treating alcohol withdrawal in the outpatient setting. (PC) |
| **Module 3. Pediatric & Adolescent Substance Misuse. 1-2 hours. Lecture or web module, video demonstration; may include skills practice and feedback.** | |
|  | **Learning Objectives:** Upon completion of this module, the learner will be able to: |
|  | Educate parents on primary prevention of substance abuse by providing emotional support, open communication and monitoring of activities. (PC) |
|  | Demonstrate use of validated screening instruments such as the CRAFFT for adolescent screening. (PC) |
|  | Educate patients, including adolescents, who are in low-risk categories in order to prevent SUDs. (PC) |
|  | Describe negative influences such as association with peers who use substances, peer pressure to use substances, etc. (PC) |
|  | Use Motivational Interviewing to encourage reductions in substance use with adolescents involved in substance use and misuse. (PC, I&CS) |
| **Module 4. Substance Abuse and the Family. 1-3 hours live. Family interview, discussion, mini-lecture or fact sheet.** | |
|  | **Learning Objectives:** Upon completion of this module, the learner will be able to: |
|  | Describe effects on the family of a member’s substance use. (MK) |
|  | Engage family members in discussions related to their involvement in and reactions to family members who use substances. (PC, I&CS) |
|  | Encourage participation in community programs such as twelve step programs that support the recovery of the family where one or more members have a SUD. (PC) |
|  | Describe ways that family members can support medical therapy such as by collaborating with medication administration. (PC) |
|  | Ask appropriate questions regarding possible SUDs-related violence. (PC, I&CS) |
|  | Identify community resources available to individuals affected by SUDs-related violence. (SBP) |
|  | Provide advice and referral to specialized treatment and community resources. (PC, I&CS) |
|  | Describe legal obligations of physicians when SUDs-related violence occurs. (PRO) |
| **Module 5. Fetal Alcohol Syndrome. 1 hour. Lecture or web module, video demonstration.** | |
|  | **Learning Objectives:** Upon completion of this module, the learner will be able to: |
|  | Describe the importance of identifying women and children at risk due to the mother’s SUD. (MK) |
|  | Describe validated screening instruments such as the TWEAK. (PC) |
|  | Provide information regarding the consequences of alcohol use during pregnancy. (PC) |
|  | Provide brief advice and counseling for moderate-to-heavy drinkers to reduce intake levels and drug users to quit using, or provide referral to community treatment services for women with SUDs. (PC, I&CS) |
|  | Provide reproductive health education about contraceptive methods, provision of contraceptive services, and client follow-up for women choosing to continue using substances.  (PC,I&CS) |
| **Module 6. Prescription Drug Abuse.** | |
| **a.** | **Appropriate prescribing practices. 1 hour. Lecture or web module.** |
|  | **Learning Objectives:** Upon completion of this module, the learner will be able to: |
|  | Demonstrate the use of formal contracts for use with patients with prescription drug abuse (e.g., when prescribing pain medications). (PC) |
|  | Continue close follow-up to determine clinician and patient follow-through with agreements in the contract. (PC,I&CS) |
|  | Describe the physician’s responsibility to prescribe medications, including controlled substances, in an appropriate manner including family members and other health care professionals. (PRO) |
| **b.** | **Outpatient management using buprenorphine. 1 hour. Lecture or web module.** |
|  | **Learning Objectives:** Upon completion of this module, the learner will be able to: |
|  | Describe possible goals for working with opioid dependent patients including abstinence from opioids and reduction in drug-related morbidity/mortality by use of a different opioid with a more favorable abuse profile. (PC, MK) |
|  | Describe methods for detoxification from opioids (MK, PC) |
|  | Describe available medications for treatment of opioid dependence including methadone, naltrexone, and buprenorphine, their indications for use, mechanisms of action, and clinical trial outcomes. (MK, PC, PBL&I) |
|  | Describe basic steps in the management of opioid dependence using buprenorphine. (MK,PC) |
| **Module 7. Common drugs of abuse such as stimulants, opioids, cocaine, amphetamines, heroin, IV drugs, marijuana, etc. 1-5 hours (one hour per drug or drug category). Case-based lecture or web module.** | |
|  | **Learning Objectives:** Upon completion of this module, the learner will be able to: |
|  | Describe and learn to recognize common drugs of abuse in the local area. (MK) |
|  | List common medical complications of one or more of these drugs. (MK) |
|  | Discuss cases seen in the clinic or hospital where drugs of abuse were involved. (MK,PC) |
|  | Describe the management of patients using drugs of abuse. (PC) |
| **Module 8. Physician impairment. 1 hour live. Presentation by physician(s) in recovery & mini-lecture or fact sheet; can also be given as lecture at resident orientation.** | |
|  | **Learning Objectives:** Upon completion of this module, the learner will be able to: |
|  | Identify the risks of health care professionals for substance misuse and resources for assistance for those impaired due to their use. (PRO) |
|  | Describe the physician’s responsibility to refer for help colleagues who are impaired due to abuse of substances. (PRO) |
|  | Participate in conversations with clinicians recovering from SUDs to learn about the difficulties they encountered because of their substance use and the challenges they faced with getting into recovery. (PRO) |
|  | Describe appropriate prescribing of medications for colleagues. (PRO,PC) |
