# Supplementary material for: Providing competency-based family medicine residency training in substance abuse in the new millennium: a model curriculum
Source: BMC Med Educ. 2010 May 11;10:33. doi: 10.1186/1472-6920-10-33 (PMC2885404; doi:10.1186/1472-6920-10-33)
Supplement: Additional file 2 — Strategies for Overcoming Substance Abuse Training Barriers. [file 1472-6920-10-33-S2.DOC]

**Additional File 2**: Strategies for Overcoming Substance Abuse Training Barriers

| **Barrier** | **Strategy** | **Module and/or Strategy** |
| --- | --- | --- |
|  | | |
| **CLINICAL BARRIERS** | | |
| **Lack of time in clinical encounters** |  | |
| 1 | Use brief screening instruments (SASQ, AUDIT-C, etc.) | Module 1b |
| 2 | Utilize brief interventions (Cutting Back, FLO charts) | Module 1c |
| 3 | Teach SBI systems which decrease MD time | Module 1f |
|  | | |
| **Stigma regarding substance abuse** |  | |
| 1 | Provide contact with recovering individuals | Modules 1e, 8 |
| 2 | Provide multiple positive faculty role models | Faculty development initiative to train at least 2 faculty from each residency |
|  | | |
| **Lack of referral resources** | Provide contact with recovering individuals & treatment center staff | Module 1e |
|  | | |
| **Confusion regarding definition of alcohol misuse** | Teach clear definitions of at-risk drinking, problem use, and alcohol dependence | Module 1a |
|  | | |
| **Skepticism regarding treatment effectiveness** | Review studies from RCT’s demonstrating effectiveness of SBIRT techniques | Module 1b |
|  | | |
| **Threats to the doctor-patient relationship** |  | |
| 1 | Develop comfortable “scripts” for use in brief advice interventions | Module 1b, 1c |
| 2 | Teach non-confrontational Motivational Interviewing techniques | Module 1c, 1d |
|  | | |
| **Low Compensation Rates** | Teach coding approaches which maximize reimbursement | Module 1f |
|  | | |
| **INSTITUTIONAL BARRIERS** | | |
| **Large percentage of residencies with no substance abuse training (25% of respondents)** | Lobby for establishment & enforcement of RRC substance abuse training requirement | Work with RRC, STFM, and AAFP |
|  | | |
| **Not enough curriculum time** |  | |
| 1 | Negotiate more time with program director |  |
| 2 | Link with other topics in medical or behavioral science curriculum (see Table 2) |  |
|  | | |
| **Limited number of trained faculty** |  | |
| 1 | Lobby for faculty development initiatives, dissemination workshops & centers of excellence | Work with NIAAA, NIDA, STFM, and HRSA |
| 2 | Collaborate with nearby faculty, ASAM members |  |
| 3 | Increase use of web-based modules |  |
|  | | |
| **Need for multiple faculty to reinforce clinical training** | Offer one-day trainings by reinstituting STFM theme days (with incentives?) | STFM, fellows of proposed faculty development project |
|  | | |
| **Lack of exposure to spectrum of substance abuse treatment** | Offer electives at treatment centers |  |
|  | | |
| **Inadequate contact with recovering patients** |  | |
| 1 | Seminars which include recovering patients | Modules 1e, 8 |
| 2 | Encourage/require AA meeting attendance | Module 1e |
|  | | |
| **Lack of acceptance of disease model** | Teach genetics and neurobiology of addition | Module 1a |
|  | | |
| **Lack of use of standardized diagnostic instruments** |  | |
| 1 | Teach use of evidence-based screening tools | Modules 1b, 3, 5 |
| 2 | Model use of tools in clinical practice & expect their use when precepting | Clinical practice/ precepting |
|  | | |
| **Lack of standards for evaluation** |  |  |
| 1 | Require observation of one substance abuse interview by faculty member | Modules 1c,1d |
| 2 | Evaluate interview using checklists [76] or validated instruments [83] | Clinical practice/ precepting |
